# Soft-bodied adaptive multimodal locomotion strategies in fluid-filled confined spaces

**DOI:** 10.1126/sciadv.abh2022

**Published:** 2021-06-30

**Authors:** Ziyu Ren, Rongjing Zhang, Ren Hao Soon, Zemin Liu, Wenqi Hu, Patrick R. Onck, Metin Sitti

**Affiliations:** 1 Physical Intelligence Department, Max Planck Institute for Intelligent Systems, 70569 Stuttgart, Germany.; 2 Institute for Biomedical Engineering, ETH Zürich, 8092 Zürich, Switzerland.; 3 Zernike Institute for Advanced Materials, University of Groningen, 9747 AG Groningen, Netherlands.; 4 School of Medicine and College of Engineering, Koç University, 34450 Istanbul, Turkey.

## Abstract

Soft-bodied locomotion in fluid-filled confined spaces is critical for future wireless medical robots operating inside vessels, tubes, channels, and cavities of the human body, which are filled with stagnant or flowing biological fluids. However, the active soft-bodied locomotion is challenging to achieve when the robot size is comparable with the cross-sectional dimension of these confined spaces. Here, we propose various control and performance enhancement strategies to let the sheet-shaped soft millirobots achieve multimodal locomotion, including rolling, undulatory crawling, undulatory swimming, and helical surface crawling depending on different fluid-filled confined environments. With these locomotion modes, the sheet-shaped soft robot can navigate through straight or bent gaps with varying sizes, tortuous channels, and tubes with a flowing fluid inside. Such soft robot design along with its control and performance enhancement strategies are promising to be applied in future wireless soft medical robots inside various fluid-filled tight regions of the human body.

## Teaser

We investigate the soft-bodied locomotion of a sheet-shaped millirobot in different fluid-filled confined spaces.

## INTRODUCTION

Untethered miniature soft robots that can minimally invasively, safely, and robustly reach and access unprecedented, risky, or hard-to-access tight body sites have great potential in future health care applications (*1*, *2*), such as targeted drug delivery (*3*), cell transplantation (*4*), endoscopy (*5*), in situ sensing (*6*), and minimally invasive surgery (*7*). These body sites are mostly physically constrained and filled with stagnant (e.g., mucus) or flowing (e.g., blood) biological fluids. Therefore, adaptive locomotion and positioning abilities in diverse confined fluid-filled spaces inside the human body are critical for future medical use of these wireless soft robots. However, designing miniature mobile robots that can adaptively navigate inside these tight fluid-filled spaces and their corresponding locomotion gaits are open issues. While changing boundary conditions and dynamic fluidic forces due to stagnant or flowing fluids result in substantial external disturbances and deviations from the predicted robot behavior, soft robots are currently designed assuming quasi-static behavior or simple boundary conditions (*8*–*11*).

To achieve robust locomotion in fluid-filled and even fluid-flowing confined spaces, a sufficient thrust force has to be generated to overcome the fluid drag and the friction with the boundaries. The conventional rigid-bodied robot designs that can achieve these functions (*12*–*14*) lack adaptability to environmental changes and may cause safety issues when interacting with soft biological tissues (*15*). One recent solution is to shrink down the robot size to be much smaller than the cross-sectional dimensions of the constrained environment to minimize the fluid drag by exploiting wall effects. One typical example of this strategy is the magnetic microroller (*16*, *17*), whose diameter is smaller than ^1^/_10_th of the smallest dimension of the blood vessel cross section. This approach, however, would not be possible when the roller size is comparable to the vessel diameter, e.g., inside capillaries.

As another recent approach, to adapt to different biological environments robustly and navigate through tight regions with opening dimensions comparable with the robot size, soft responsive materials can be used to build the robot body to enable soft-bodied multimodal locomotion (*18*). This strategy has enabled the robots to pass through a dry confined region or to be passively carried by the fluid flow in a bending tube (*19*). However, in these two works (*18*, *19*), active soft-bodied locomotion and maneuverability in fluid-filled confined spaces have not been demonstrated, and the influence of different factors of the fluid-filled confined environments on the soft-bodied locomotion is not understood well.

**Fig. 1:**
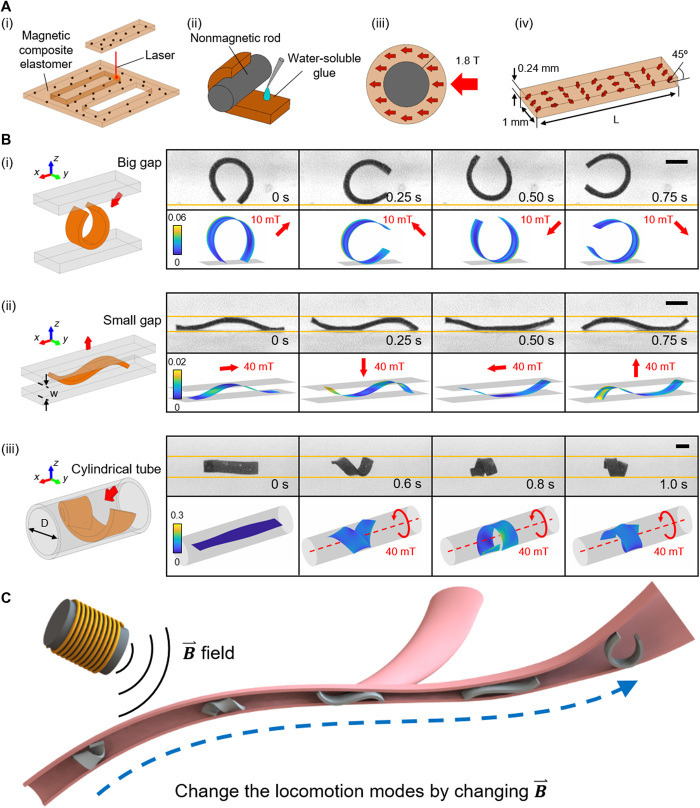
Fabrication of the sheet-shaped magnetic soft millirobot and its main deformation and locomotion modes in different fluid-filled confined environments. (**A**) The soft robot fabrication process. (i) The robot is cut from a magnetic composite elastomer film using laser cutting. (ii) The robot is wrapped to a nonmagnetic cylindrical rod with the help of water-soluble glue. (iii) The robot is put in a uniform 1.8-T magnetic field for magnetization. (iv) The dimensions and magnetization profile of the robot. (**B**) Different body deformations are optimal in different confined spaces. (i) In a big gap (δ = 1.1), the robot can curl into a C shape when $\overset{⃑}{B}$ is in the *x*-*z* plane. (ii), In a small gap (δ = 7.33), the robot can deform into the sinusoidal shape when $\overset{⃑}{B}$ is in the *x*-*z* plane. (iii) In a cylindrical tube (γ = 3.44), the robot can deform into a helical shape when $\overset{⃑}{B}$ is in the *y*-*z* plane. The finite element–based simulations can predict the robot body deformation modes in given boundary conditions. The red arrows indicate the direction of $\overset{⃑}{B}$ at that time instant. The colormap indicates the equivalent von Mises strain. The experimental environments are filled with a viscous fluid (η = 720 cSt). Scale bars, 1 mm. (**C**) Conceptual schematic depicting the adaptive multimodal locomotion of the sheet-shaped robot in various confined spaces with varying cross-sectional geometries and sizes. Photo Credits: Ziyu Ren, Max Planck Institute for Intelligent Systems.

In nature, small-scale soft-bodied organisms can adapt to changing environments and achieve effective locomotion through adaptive body-environment interactions (*20*–*22*). For instance, *Caenorhabditis elegans* can use the interplay between its active muscle control and the environmental conditions to realize various adaptive undulatory locomotions (*20*). Inspired by these adaptive soft body–environment interactions of small-scale soft-bodied organisms, here we demonstrate that the environments in fluid-filled confined spaces provide opportunities to achieve previously unrealized soft robot locomotion. Different adaptive locomotion modes can be realized by concurrently exploiting the surrounding boundaries, the hydrodynamic and frictional forces, and the robot’s active soft-bodied deformation. More specifically, we propose a series of control strategies for magnetically programmed sheet-shaped soft robots (Fig. 1Aiv) to change their locomotion modes to accommodate different environmental conditions in various confined spaces filled with stagnant or flowing fluids, in compared to our previous work realizing locomotion in dry confined regions only (*18*). As another novelty, this work includes detailed computational simulations and characterization of the soft robot’s fluid-filled channel propulsion using single- and new multiwave body undulation and new helical locomotion modes for our improved understanding. In gaps that are much larger than the robot size, the robot can realize rolling locomotion by curling into a circle (Fig. 1Bi). In smaller gaps, the robot can conduct body undulation–based (undulatory) crawling or swimming (Fig. 1Bii). In cylindrical tubes, the robot can achieve helical surface crawling and even withstand dynamic fluid flows (Fig. 1Biii). Each locomotion mode has its unique advantages. For example, undulatory crawling can easily go through a highly bent path with a small gap. Undulatory swimming can enable a rapid passage through a straight slit. Helical surface crawling can enable propulsion with and against fluid flows and withstand the flow even when the actuation is turned off.

Investigation of the physical mechanisms behind these locomotion modes in this study reveals that the undulatory crawling and helical surface crawling modes rely on the friction between the robot body surface and the surrounding boundaries for efficient and robust propulsion. The undulatory swimming mode relies on transporting the fluid to produce the thrust force. On the basis of this knowledge, we further propose different performance enhancement strategies to increase the robot locomotion speed and maneuverability or enhance its capability to withstand the opposing fluid flows. All the proposed locomotion modes can be achieved through a single soft robot design. As a demonstration, the robot is navigated to pass through a phantom, mimicking the Eustachian tube, using these multiple locomotion modes. The proposed control and performance enhancement strategies make the sheet-shaped robot promising to be deployed in various lumens of the human body in the future.

## RESULTS

### Adaptive deformation of the robot’s soft body in various confined spaces

The sheet-shaped soft robot with a sinusoidal magnetization profile (Fig. 1Aiv) was studied here because of its versatility in producing multimodal soft-bodied locomotion, specifically its capability to generate undulatory body waves in confined spaces (*18*). To program the sinusoidal magnetization profile along the robot length, the robot was cut from a thin film of the magnetic composite elastomer, wrapped to a nonmagnetic cylindrical rod with the help of water-soluble glue, and lastly magnetized in a 1.8-T uniform magnetic field (Fig. 1A, i to iii). The ferromagnetic neodymium-iron-boron (NdFeB) microparticles tend to align their magnetization directions along with the external magnetic field $\vec{B}$, inducing torques that deform the soft elastomeric robot body (*8*, *23*). The sheet-shaped robots developed in this study had the same width (1 mm) and thickness (0.24 mm) and similar sinusoidal magnetization profiles with an initial phase shift of 45° (Fig. 1Aiv). The robot lengths were designed to be at least 11 times larger than the robot thickness to achieve large deformations (fig. S1).

Although the magnetization profile along the robot length was fixed, the robot could still achieve different deformation modes to adapt to different environments using different external magnetic field control inputs. When the robot is in a big gap, where the ratio δ between the robot length and the gap width (δ = *L*/*w*) is smaller than 2.6 (fig. S2), applying $\vec{B}$ in the *x*-*z* plane can deform it into a “C” shape. Its orientation is determined by the direction of $\vec{B}$ (Fig. 1Bi). When the robot comes into a small gap, where δ>2.6, applying $\vec{B}$ in the *x*-*z* plane no longer produces the C shape, while a sinusoidal shape appears because of the squeezing of the upper and lower boundaries. The positions of the wave crests and troughs are determined by the direction of $\vec{B}$ (Fig. 1Bii). When the robot navigates into a cylindrical tube, which has an inner diameter larger than the robot width, applying a rotating $\vec{B}$ in the *z*-*y* plane can twist it into a helical shape (Fig. 1Biii). By using these shape changes, the robot can achieve adaptive locomotion in confined spaces with different cross-sectional geometries and sizes (Fig. 1C). In the following, detailed analyses on undulatory crawling, undulatory swimming, and helical surface crawling are conducted through systematic experiments and simulations. For the ease of discussion, the detailed conditions of each experiment are provided in table S1 and will not be reiterated in the main text.

### Undulatory crawling and swimming locomotion modes

**Fig. 2:**
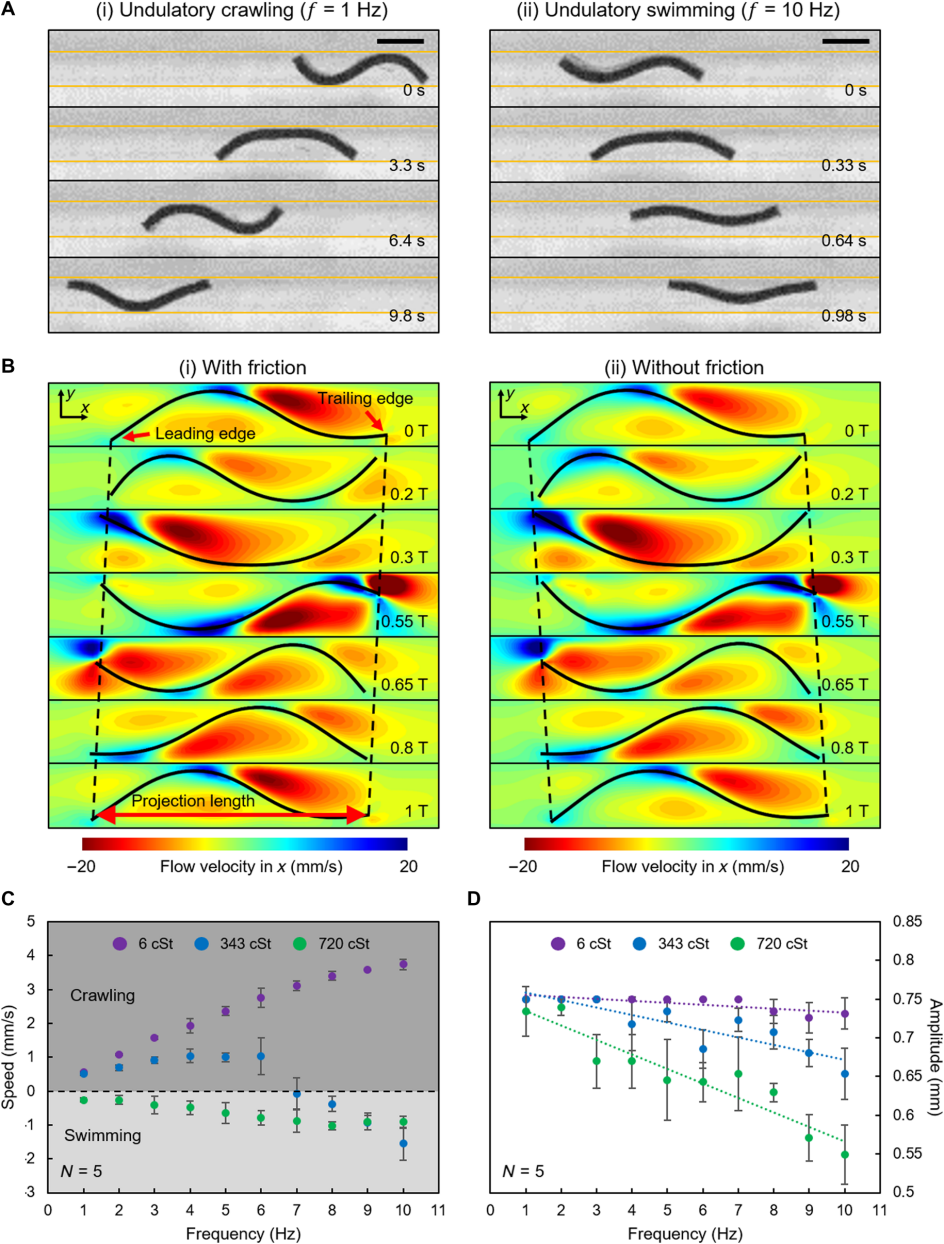
Undulatory crawling and swimming locomotion modes inside fluid-filled channels. (**A**) Experimental transition of the propulsion direction of the robot’s undulating body by changing the magnetic actuation frequency. (i) The sheet-shaped robot performs undulatory crawling when the actuation frequency is 1 Hz. (ii) The sheet-shaped robot performs undulatory swimming when the actuation frequency is 10 Hz. The yellow lines indicate the position of the boundaries. Scale bars, 1 mm. (**B**) The simulation of the undulatory locomotion modes. The simulated robots were actuated at ∣$\overset{⃑}{B}$∣ = 40 mT, *f* = 5 Hz. (i) The simulated robot performed undulatory crawling when the friction was considered. (ii) The simulated robot performed undulatory swimming when the friction was removed. The black dashed lines indicate the displacement along the *x* direction. (**C**) Frequency sweeping experiments for three different fluid viscosities. (**D**) The variation of the undulatory amplitude for three different fluid viscosities. The trendline is fitted by the least-square method. In (C) and (D), *N* is the number of trials in each case. The error bars stand for the error of the mean. Photo Credits: Ziyu Ren, Max Planck Institute for Intelligent Systems.

By applying a rotating $\vec{B}$ in the *x*-*z* plane, the sheet-shaped robot produced traveling waves along the body’s longitudinal direction and achieved two distinct locomotion modes in a small gap filled with viscous fluid (η = 343 cSt). As the experiments show in Fig. 2A and movie S1, a rotating $\vec{B}$ with a frequency of 1 Hz resulted in locomotion in the same direction as the traveling body wave of the robot. However, keeping the magnitude and the rotating direction of $\vec{B}$ fixed while increasing the actuation frequency to 10 Hz reversed the robot locomotion direction.

To understand when and why such transition in the locomotion direction occurred, we carried out numerical simulations (see Materials and Methods: “Numerical simulations” section; fig. S3A). In the first simulation, we considered both the fluid-structure interaction and the friction between the robot and the channel sidewalls. Under these conditions, the robot was found to move in the −*x* direction, i.e., in the same direction as the body wave transmission. A close inspection revealed that such movement was achieved by the robot through cyclically changing the projection length (fig. S4A) while alternatively anchoring both edges of the body to the channel walls (Fig. 2Bi). From 0 to 0.2 T (T denotes one period), the trailing edge detached from the bottom wall and moved to the upper wall, while the anchoring between the leading edge and the bottom channel wall was well established. Therefore, the robot could contract the body projection length by transmitting the body wave in the −*x* direction. This motion caused the forward translation of the trailing edge. From 0.2 to 0.3 T, the robot firmly anchored its trailing edge to the upper channel wall, while its leading edge detached from the bottom wall and moved to the upper wall. During this motion, the robot was able to stretch its projection length by transmitting the body wave in the −*x* direction while being attached to the upper wall at its trailing edge. This motion caused the forward translation of the leading edge. From 0.3 to 0.55 T, there was no change in the projection length, leading to negligible translation of both edges. In the remaining period of the cycle, the robot repeated the contraction (0.55 to 0.65 T) and stretching (0.65 to 1 T) of the body projection length. By cyclically contracting and stretching the body projection length while being attached at the leading and trailing edge, respectively, the robot produced a pronounced net displacement in the −*x* direction. Such concertina-like propulsion can be observed in limbless animals such as snakes (*24*), and we refer to this locomotion mode as undulatory crawling in this paper.

If we removed the friction acting on the simulated robot, then the robot started to swim in the opposite direction (Fig. 2Bii) as it was unable to anchor to the walls and hold its current position during the undulatory motion. The thrust force was obtained by creating a net flow, quantified by normalized net flow (Materials and Methods: “Numerical simulations” section; fig. S4B), in the same direction as the body wave transmission (i.e., in the −*x* direction), resulting in propulsion in the +*x* direction. We refer to this locomotion mode as undulatory swimming here. The undulatory crawling and swimming modes have opposite locomotion directions, which manifests a competition between the two modes, i.e., enhancing one of these modes would inevitably weaken the other.

When the robot is actuated at different frequencies or placed in different fluid viscosities, it is subjected to different friction and hydrodynamic forces. Increasing the fluid viscosity or the actuation frequency will decrease its undulation amplitude and consequently decrease its friction with the walls. When increasing the fluid viscosity, the undulation amplitude decreases because of the increase in the fluid drag that acts as the external damping. When increasing the actuation frequency, the undulation amplitude decreases because of the increase in the fluid drag and the internal damping induced by the material viscoelasticity (*25*). Thus, the transition between the undulatory swimming and crawling modes is largely dictated by the fluid viscosity and the rotating frequency of $\overset{⃑}{B}$. To verify this hypothesis, we fixed δ = 4.67 and experimentally measured the locomotion speeds and the undulation amplitudes of the robot at frequencies ranging from 1 to 10 Hz in fluids with three different viscosities (Fig. 2, C and D). The undulation amplitude was determined by averaging the amplitudes measured from four typical waveforms as shown in Fig. 2A. At the lowest fluid viscosity (η = 6 cSt), the robot only performed undulatory crawling throughout the whole frequency range, and the undulation amplitude only showed a slight drop after 8 Hz. At an intermediate fluid viscosity (η = 343 cSt), the robot performed undulatory crawling below 7 Hz and transitioned to undulatory swimming above 7 Hz at Reynolds number (*Re*) ranging from 0.5 × 10^−3^ to 5.4. The transition appeared because the undulation amplitude gradually decreased with increased frequency. During this process, the anchoring of the leading and trailing edges was gradually weakened, and the hydrodynamic propulsion gradually dominated. At the most viscous fluid (η = 720 cSt) case, the robot only performed undulatory swimming throughout the whole frequency range at *Re* ranging from 1.1 × 10^−3^ to 3.2, and the undulation amplitude decreased rapidly with the increased frequency. Because the undulation amplitudes at high frequencies (*f* >8 Hz) became very small, the peak swimming speed was found to be even smaller than that achieved in the intermediate fluid viscosity.

### Locomotion speed enhancement of the two undulatory locomotion modes

**Fig. 3:**
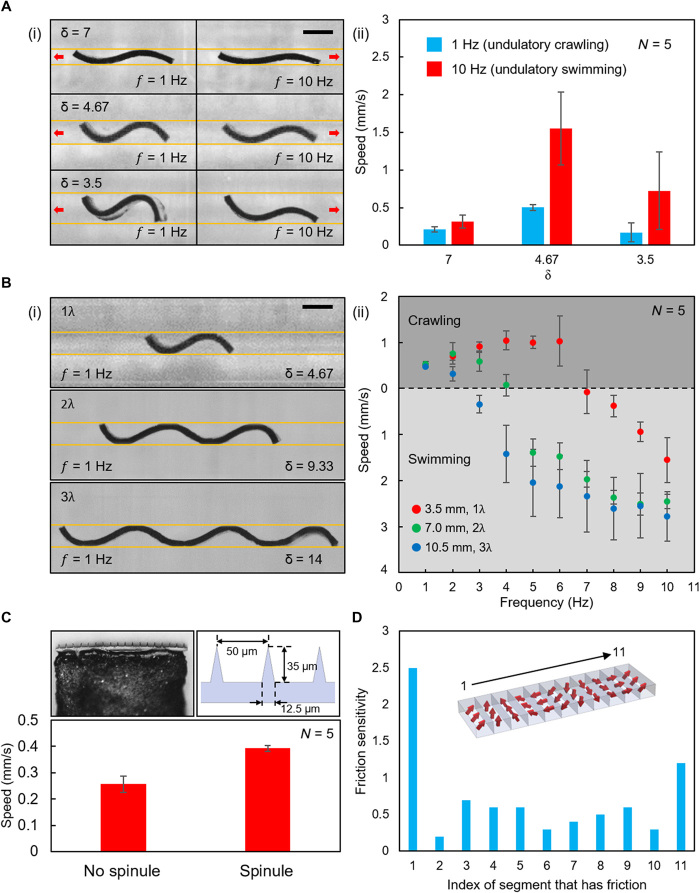
Different factors influencing the speed of the undulatory locomotion modes in experiments. (**A**) The influence of δ on undulatory locomotion speed. (i) The deformation of the robot at different δ during the crawling and swimming locomotion modes. The red arrows indicate the propulsion directions. (ii) The average speeds in different cases. (**B**) The influence of creating multiwavelength sinusoidal magnetization profiles. (i) The deformation of the robot with multiwavelength sinusoidal magnetization profiles. (ii) The average speed as a function of frequency for different robot lengths and magnetic fields. (**C**) The influence of the microspinules on undulatory crawling speed. The microscopic photo and dimensions of the spinules are shown in the small insets. (**D**) Simulation analysis on the friction sensitivity of each part of the robot body. In (A) and (B), the yellow lines indicate the position of the boundaries. In (A) to (C), *N* is the number of trials in each case. The error bars stand for the error of the mean. Scale bars, 1 mm. Photo Credits: Ziyu Ren, Max Planck Institute for Intelligent Systems.

To achieve high undulatory locomotion speed, the robot should be designed to have an appropriate δ. In the experiments shown in Fig. 3A and movie S2, δ was tuned by changing the channel width (*w* = 0.5, 0.75, and 1.0 mm) while fixing the robot length (*L* = 3.5 mm). The fluid in the channel had a viscosity of 343 cSt so that the robot could crawl at 1 Hz and swim at 10 Hz. At δ = 7, only a small deformation amplitude could be achieved because the narrowly spaced channel walls overly constrained the robot’s deformation. Compared to the undulatory locomotion at δ = 4.67, the smaller amplitude decreased the amount of contraction and stretching of the projection length during crawling and the amount of fluid transported during swimming. Hence, both the crawling and swimming speeds dropped. At δ = 3.5, the robot could hardly anchor its leading and trailing edges during crawling. Hence, the crawling speed decreased compared to the case of δ = 4.67. The widely spaced channel walls also influenced the body kinematics during swimming. In this case, the amplitude of the front part of the body became very small, which decreased the swimming speed. More extensive parameter sweeping experiments, in which η, *f*, and δ were systematically changed, further show that the robot can achieve the highest undulatory crawling or swimming speed with δ at around 4 to 4.67 at the current experimental conditions (fig. S5).

The undulatory swimming speed can be enhanced by connecting multiple short robots in series to create multiwavelength (i.e., multiperiod) sinusoidal magnetization profiles (Fig. 3Bi, fig. S6A, and movie S3; Materials and Methods: “Soft millirobot fabrication” section). The robot’s multiple wavelength did not significantly change the capabilities in anchoring, contraction, and stretching during the crawling. Hence, the crawling speeds at low actuation frequencies (*f* = 1 Hz) did not show a significant difference among the robots with different waveform numbers in experiments [Fig. 3Bii; analysis of variance (ANOVA), *F*(2,12) = 2.297, *P* = 0.143]. However, the capability to transport fluid was enhanced by applying this strategy. This can be seen from the decrease of the transition frequency and the increase of the peak swimming speed (Fig. 3Bii). In addition, creating multiple waveforms also makes it possible to achieve effective locomotion at a very large δ.

In the case that the confined space is covered by soft tissues, the undulatory crawling speed can be enhanced by integrating microspinules at both edges of the robot (fig. S6B). In this way, the anchoring of the leading and the trailing edges can be improved by spinule friction (*26*, *27*). The improvement brought by the microspinules can be verified by experimentally comparing the speeds of two single-waveform robots crawling at δ = 4.67, and η = 720 cSt, in which one of the robots had spinules at both ends while the other one did not (Fig. 3C). However, if the channel walls are hard and smooth, then the spinule would not significantly contribute to the friction enhancement because of the lack of interlock between the spinules and the contact surface (*26*, *27*).

The position where the friction should be enhanced can also influence the undulatory crawling speed. In the simulations, we separated the simulated robot into 11 equal-length segments and imposed a friction coefficient to one of the segments while keeping others smooth. The variations of the crawling speeds with different friction coefficients and friction enhancement locations are shown in fig. S7. The friction sensitivity, which is defined as the slope of the linear fitted line of the speed data points, quantifies how efficient it is to increase the crawling speed by increasing the friction coefficient at that location. The simulation results revealed that increasing the friction at both ends is the most efficient way to enhance the undulatory crawling speed, although increasing friction in the middle regions could also slightly increase the crawling speed (Fig. 3D). This result justified the position that we chose to integrate the spinules.

### Maneuverability enhancement of the undulatory crawling mode

**Fig. 4:**
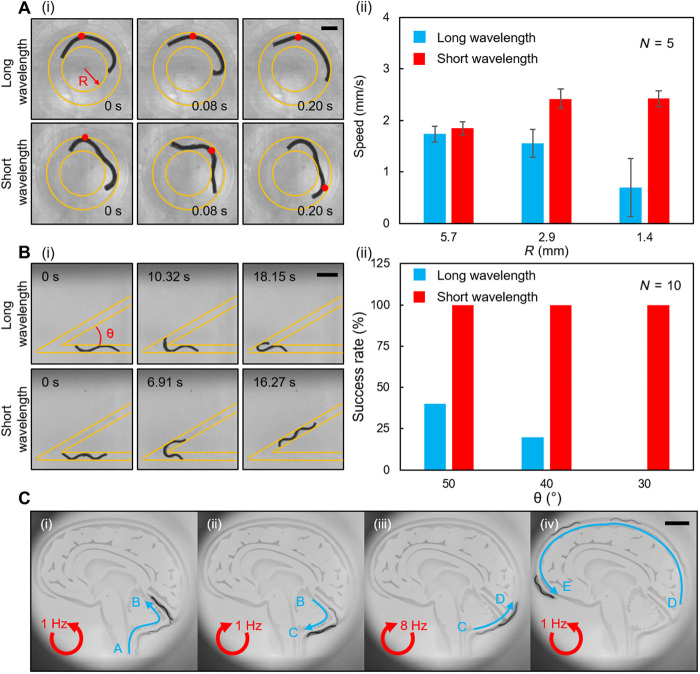
Experimental maneuverability of the soft robot performing the undulatory crawling mode. (**A**) The influence of the undulation wavelength on the ability to pass a circularly curved gap. (i) The robot with a shorter wavelength can better transmit the body wave. (ii) The comparison of the crawling speeds of the robots with different wavelengths at different *R*. Photo Credit: Ziyu Ren, Max Planck Institute for Intelligent Systems. (**B**) The influence of the wavelength on the ability to take a sharp turn. (i) The robot with a longer wavelength fails to anchor its leading edge to the new branch, while the robot with a shorter wavelength succeeds. (ii) The comparison of the success rates of the robots with different wavelengths at different θ. Photo Credit: Ren Hao Soon, Max Planck Institute for Intelligent Systems. (**C**) Steering the sheet-shaped robot in a phantom mimicking the brain aqueduct by tuning the magnetic rotation frequency. The directions of rotating magnetic field control inputs are indicated by red arrows. Photo Credit: Ren Hao Soon, Max Planck Institute for Intelligent Systems. In (A) and (B), the yellow lines indicate the position of the boundaries. *N* is the number of trials in each case. The error bars stand for the error of the mean. Scale bars, 1 mm (A), 2 mm (B), and 5 mm (C).

In terms of going through highly curved small gaps or taking sharp turns, undulatory crawling is superior to undulatory swimming because a large body bending is required in these two cases. When the robot’s length is fixed, the robot’s maneuverability is largely influenced by the body undulation wavelength. To investigate how the body wavelength influences the capability of passing a curved gap when the robot length is fixed, we prepared two robots with an identical length of 5.5 mm, while one of them had a two times shorter wavelength than the other one. The robot with a shorter wavelength was fabricated by serially connecting two shorter robots that had lengths of 2.75 mm and one-period magnetization profiles (Fig. 4Ai, fig. S6A, and movie S4; Materials and Methods: “Soft millirobot fabrication” section). We experimentally tested the robots in three ring-shaped channels with different radii (*R* = 1.8, 3.2, and 6.1 mm) and revealed that the robot with a shorter body wavelength performed better in terms of the crawling speed (Fig. 4Aii). The superiority of the short-wavelength design became more obvious when the curvature of the channel became larger. By comparing the deformation of these robots in the ring-shaped channel with the largest curvature (*R* = 1.8 mm), we found that the robot with a longer wavelength could hardly produce a body traveling wave, which was not the case of the robot with a shorter wavelength. The inability to produce the body traveling wave impeded the establishment of the concertina-like cyclic locomotion. As a consequence, the crawling speed dropped.

A shorter wavelength can also help the robot take a sharp turn, which was demonstrated in the experiments shown in Fig. 4B and movie S4. Three channels with different sharp turning angles (θ = 50°, 40°, and 30°) were prepared. The robots were tested in each channel 10 times, and the success rate to go through the turning angle was calculated. The robot with a longer wavelength had few chances to go through the sharp angle, while the robot with a shorter wavelength could take the sharp turn in each trial. Compared to the long-wavelength robot, the short-wavelength robot had more contact points to the sidewalls, which helped the robot to adjust its body orientation and to avoid being stuck at the angle vertex.

By simply tuning the actuation frequency and direction, it is possible to steer the robot at a bifurcated point and achieve the on-demand selection of the right branch. A low actuation frequency induces a large undulation amplitude and allows the robot to take the sharp turning, while a high actuation frequency produces a small undulation amplitude and makes the robot tend to move along the original direction. To demonstrate this concept, we tested the robot in a branched channel that mimicked the shape of the human brain cerebral aqueduct (Fig. 4C and movie S4). The channel width was designed to make δ range from 4.12 to 7. Because the channel was highly bending, we used a robot that had two waveforms. By applying the counterclockwise rotating $\vec{B}$, the robot crawled from position A into lateral branch B by taking a sharp turn at the bifurcation point (Fig. 4Ci). Then, the rotating direction of $\vec{B}$ was reversed to withdraw the robot back to the main branch (Fig. 4Cii). To avoid entering the lateral branch again and keep moving in the main branch, we increased the rotating frequency of $\vec{B}$ to 8 Hz to decrease the robot’s beating amplitude so that the leading edge of the robot could not anchor to the lateral branch. Therefore, the robot kept inside the main branch and swam from position C to position D (Fig. 4Ciii). After bypassing the lateral branch, $\vec{B}$ was switched back to be 1 Hz and counterclockwise, and the robot could go through the rest of the passage (Fig. 4Civ).

### Helical surface crawling locomotion mode

**Fig. 5:**
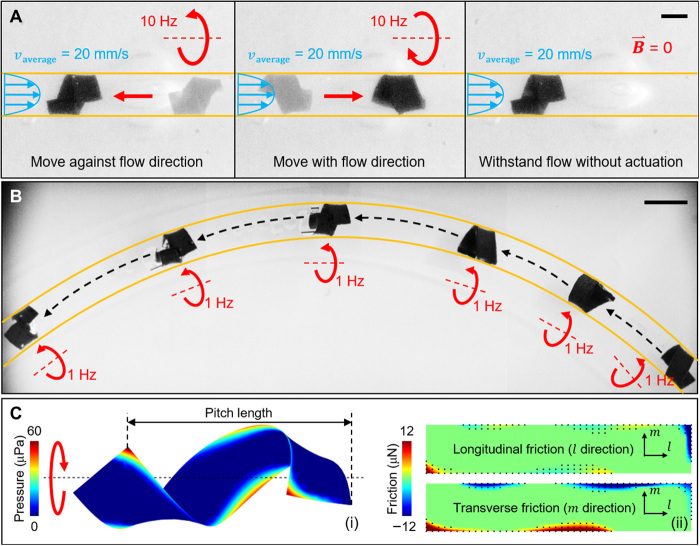
Helical surface crawling locomotion mode in experiments and simulation. (**A**) As a new locomotion mode of the sheet-shaped robot, the robot can move against or with the fluid flow by helical surface crawling inside a cylindrical tube. It can also withstand flow when $\overset{⃑}{B}$ is off. The blue arrows indicate the direction of the fluid flow. The fluid filled in the tube is water here. (**B**) The robot can crawl in a bending tube by controlling the direction of the rotating axis of $\overset{⃑}{B}$. (**C**) Simulation analysis on (i) distribution of the contact pressure and (ii) the anisotropic distribution of friction. In (ii), the rectangles represent the unwrapped sheet-shaped robot. The black dots indicate the positions of the contact area under the simulation settings. In (A) and (B), the yellow lines indicate the positions of the boundaries. The directions of the rotating magnetic field control inputs are indicated by red arrows. Scale bars, 1 mm (A) and 2 mm (B). Photo Credits: Ziyu Ren, Max Planck Institute for Intelligent Systems.

When the robot moves inside a fluid-filled cylindrical tube, simply providing a rotating $\vec{B}$ in the *x*-*z* plane always fails to produce stable undulatory locomotion. This is because the robot tends to rotate along the *x* axis of the tube under the external rotating $\vec{B}$ (fig. S8). Stable locomotion in a cylindrical tube can be achieved by changing the plane of the rotating $\vec{B}$ from the *x*-*z* plane to the *y*-*z* plane to twist the flat robot into a helical shape as shown in Fig. 1Biii. After forming the helical shape, the robot can maintain this configuration and rotate along with the rotating $\vec{B}$. One merit of such helical surface crawling is that the robot body does not block the tube so that the fluid can still pass through, which is a critical feature for safely navigating inside blood vessels and other tubular regions inside the body without blocking the blood or other biological fluid flows. This feature also endows the robot with the capability to move with or against the fluid flow and allows the robot to anchor to a position to withstand the flow even when $\vec{B}$ is off, which outperforms the undulatory locomotions (fig. S9). These capabilities are shown in Fig. 5A and movie S5. In addition to the helical surface crawling in a straight cylindrical tube, the robot can also be steered in a bending tube by dynamically aligning the direction of the rotating $\vec{B}$ to the tangential direction of the tube, as shown in Fig. 5B and movie S6.

The propulsion of the rotating helically shaped robot was achieved because of the anisotropic friction force distribution along the body (Fig. 5C). The simulation of a rotating helical robot (fig. S3B) revealed that only part of the robot surface touching the tube wall, inducing a heterogeneous pressure distribution. As a result, the friction along the longitudinal (***f***_//_) and transverse (***f***_⊥_) directions still showed differences even if the frictional coefficient between the robot and tube wall was isotropic. The difference between ***f***_//_ and ***f***_⊥_ contributed to the thrust force during this helical surface crawling locomotion (Materials and Methods: “Force analysis of helical surface crawling” section; fig. S10).

### Locomotion performance enhancement of the helical surface crawling mode

**Fig. 6:**
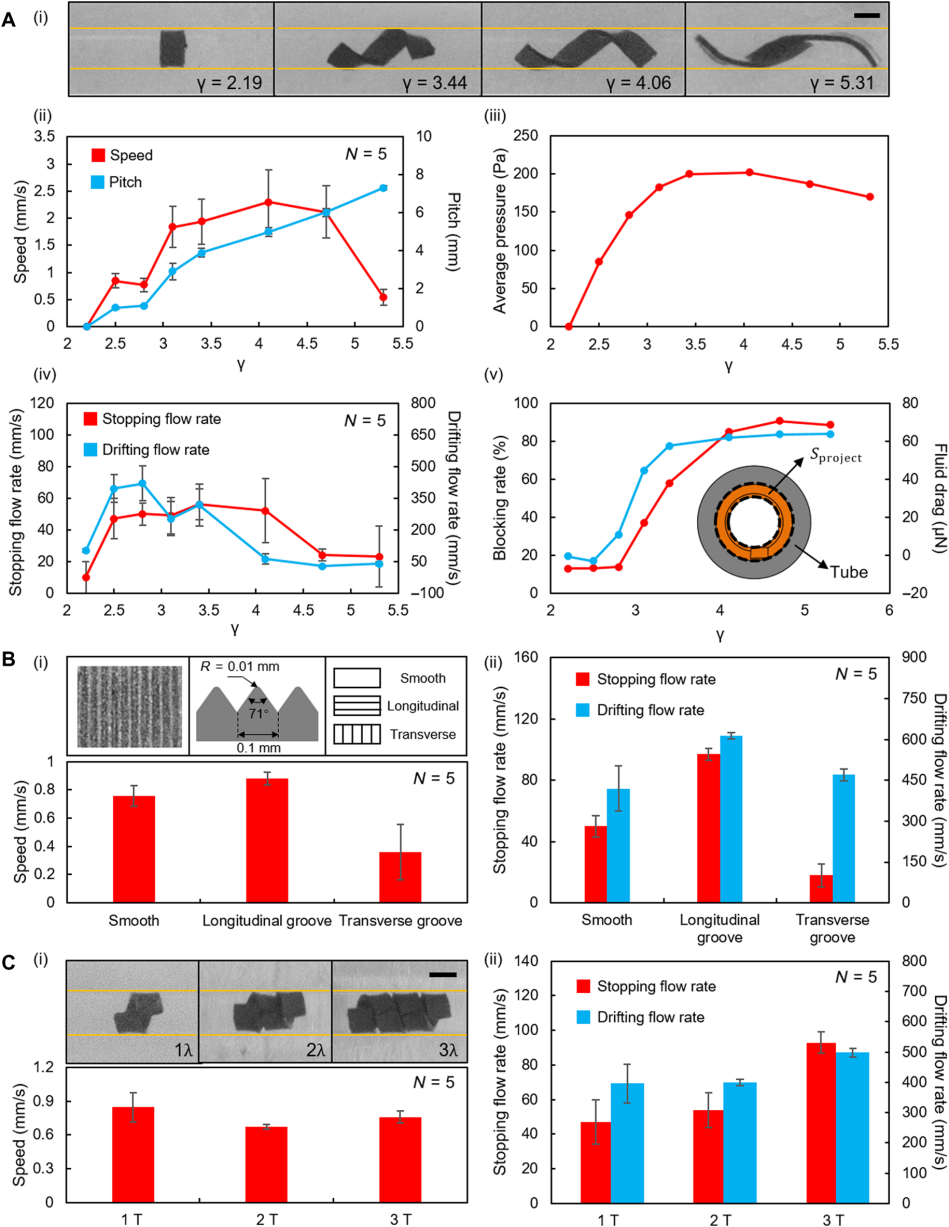
The factors that influence the helical surface crawling performance in experiments. (**A**) The influence of γ on helical surface crawling performance. (i) The deformation of the robot at different γ. (ii) The pitch length and crawling speeds achieved at different γ. (iii) Simulation results of the variation of the average pressure with varying γ. (iv) The stopping and drifting flow rates achieved at different γ. (v) Simulation analysis of the influence of γ on the blocking ratio and the fluid drag. (**B**) The influence of the robot surface grooves on helical surface crawling performance. (i) The crawling speeds achieved at different groove orientations. The optical microscope image and dimension and orientation schematics of the grooves are shown in the small insets. (ii) The stopping and drifting flow rates achieved by different grooved patterns. (**C**) The influence of creating multiwavelength sinusoidal magnetization profiles. (i) The crawling speeds obtained at different numbers of periods show no significant difference [ANOVA, *F*(2,16) = 2.381, *P* = 0.124]. The helical shapes formed by the robots with multiwavelength sinusoidal magnetization profiles are shown in the small insets. (ii) The stopping and drifting flow rates at different numbers of periods. In (A) to (C), the yellow lines indicate the position of the boundaries. *N* is the number of trials in each case. The error bars stand for the error of the mean. Scale bars, 1 mm. Photo Credits: Ziyu Ren, Max Planck Institute for Intelligent Systems.

The locomotion performance of the helical surface crawling is greatly influenced by the geometry of the helical shape that is quantified by its pitch length (Fig. 5Ci) and can be varied by changing γ (=*L*/*D*), which is the ratio between the robot length (*L*) and the inner tube diameter (*D*) (movie S7). In the experiments shown in Fig. 6A, we fixed the tube inner diameter to be 1.6 mm and varied the robot length from 3.5 to 8.5 mm to render a series of γ from 2.19 to 5.31. The achieved pitch lengths and the crawling speeds were measured in each case (Fig. 6Aii). When γ = 2.19, the robot would curl into a ring shape with zero pitch length, and no translational motion occurred. If we increased γ from 2.5 to 4.06, then the pitch length increased almost linearly except at the data point where γ = 2.81. The variation of the crawling speed also followed this trend. The increase in crawling speed can be attributed to the increase in the pitch length and the average pressure against the tube, which is shown by simulations conducted in the same conditions (Fig. 6Aiii). The average pressure was calculated by averaging the pressure of the robot’s contact surface. The increase in pitch length increased the possible maximum translation distance per rotation, and the increase in average pressure increased the friction force that is the source of the thrust ***f****_x_* (Materials and Methods: “Force analysis of helical surface crawling” section; fig. S10). The speed reached a peak at γ = 4.06. If γ was further increased, then the crawling speed dropped. This is because further increasing γ caused a decrease in the pressure and hence a decrease in ***f****_x_*. More extensive parameter sweeping experiments, in which *f* and δ were systematically tuned, further demonstrate that the robot achieved the highest helical surface crawling speed at γ = 4.06, *f* = 6 Hz at the current experimental conditions (fig. S11).

Apart from the crawling speed, γ also influences the robot capability to withstand the fluid flow, which was quantified by the stopping flow rate and the drifting flow rate measurements. The stopping flow rate is the minimum average flow rate that prevents the forward motion of the robot actuated at 3 Hz. The drifting flow rate is the minimum average flow rate that flushes the robot away from its anchoring position when $\overset{⃑}{B}$ is off. The experimental results are shown in Fig. 6Aiv. In general, the drifting flow rate was higher than the stopping flow rate of a specific robot design. This is due to the fact that the friction between two polymer surfaces drops when sliding happens (*28*). This feature enabled the robot to remain anchored within the flowing fluid for long-duration operations. Although the robot performed the best in terms of the crawling speed when γ = 4.06, it did not achieve the highest stopping and drifting flow rates at this γ. This was due to the fact that the fluid drag markedly increased when γ increased from 2.19 to 4.06, which was caused by the increase in the blocking ratio, defined as the ratio between *S*_project_ and the cross-sectional area of the tube (Fig. 6Av and fig. S12). The maximum average pressure and the minimum fluidic drag appear at different γ values, and the best compromise was achieved at around γ = 2.81 according to Fig. 6Aiv.

The critical role of friction in helical surface crawling motivated us to modify the robot surface for higher propulsion speeds. To this end, we created grooves on the robot surface (Materials and Methods: “Soft millirobot fabrication” section) for the purpose of draining the liquid from the contact area to establish reliable solid-solid contact (*29*, *30*) and enhancing the frictional anisotropy (*31*–*34*). We created two types of grooves that oriented longitudinally or transversely (Fig. 6Bi) to induce different friction anisotropies and increase the overall friction (fig. S13B). If the grooves were created longitudinally to increase more lengthwise friction (***F***_∥_), then the crawling speed, the stopping flow rate, and the drifting flow rate all increased compared with the smooth robot in experiments (Fig. 6B, i and ii). However, if the grooves were created transversely to increase more widthwise friction (***F***_⊥_), then the crawling speed and the stopping flow rate all dropped compared with the smooth robot. Creating longitudinal grooves along the robot did improve the overall performance of the helical surface crawling, although higher magnetic actuation torques were needed (Materials and Methods: “Force analysis of helical surface crawling” section; table S1). A rotating $\vec{B}$ with magnitude of 40 mT and a frequency of 3 Hz could rotate a smooth 4.5-mm robot in an elastomeric tube with an inner diameter of 1.6 mm, while it failed to rotate a grooved robot at the same experimental conditions. Therefore, a higher $\vec{B}$ with a magnitude of 45 mT was applied to rotate the grooved robot at a frequency of 3 Hz.

Another performance enhancement strategy is to connect multiple robots in series to create a robot with multiwavelength sinusoidal magnetization profiles (Fig. 6Ci). In this case, the pitch lengths and crawling speeds did not show a significant statistical difference when compared to the single-wavelength robot in experiments [ANOVA, *F*(2,16) = 2.381, *P* = 0.124], demonstrating that the pressure distribution on each single-wavelength segment did not change too much. In addition, the increase in the number of segments increased the contact area without changing the blocking ratio. Consequently, the friction increased while the fluid drag remained unchanged at the same flow conditions. This fact enabled the robot to withstand higher fluid flows (Fig. 6Cii).

### Adaptive locomotion in a fluid-filled confined space with varying cross-sectional geometries and sizes

**Fig. 7:**
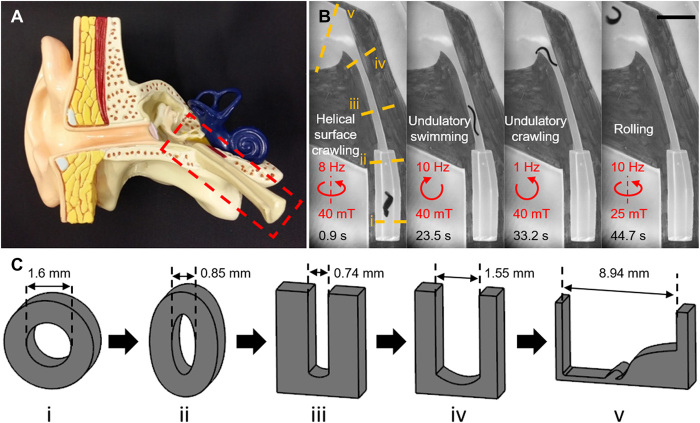
Navigating the soft robot inside the Eustachian tube phantom with diverse cross-sectional geometries and sizes filled with a shear-thinning fluid using its four different locomotion modes. (**A**) Anatomical model of the human ear. The red dashed box indicates the Eustachian tube that is mimicked by the simplified phantom. (**B**) The robot can adapt to the local environmental constraints and conditions of the phantom by changing its locomotion mode to helical surface crawling, undulatory swimming, undulatory crawling, and rolling. The red arrows indicate the direction of $\overset{⃑}{B}$. Scale bar, 5 mm. (**C**) The schematic of the diverse cross-sectional geometries and sizes in this phantom. Photo Credits: Ziyu Ren, Max Planck Institute for Intelligent Systems.

By tuning the magnetic field according to the local environmental conditions, the sheet-shaped soft millirobot can adaptively locomote in confined spaces with varying cross-sectional geometries and sizes that are commonly observed inside the human body (*35*–*37*). As a proof-of-concept demonstration, the robot was tested in a phantom that mimicked the Eustachian tube connecting the throat and the middle ear (Fig. 7A and movie S8). The phantom started from a tubular space that gradually tapered to render an elliptical cross-sectional shape. Following the tubular space was a slit-like gap that gradually increased in width. The outlet of the phantom was a large space that mimicked the tympanic cavity. In some diseases, such as the upper respiration infection, the fluid may accumulate at the middle ear space, and otitis media with effusion occurs (*38*). To mimic such environment, the phantom was filled with a shear-thinning non-Newtonian fluid that has comparable properties as the middle ear effusion (fig. S14B) (*39*, *40*). To navigate the robot through the phantom, we applied different control signals (Fig. 7B). When the robot was in the circular tube, an 8-Hz rotating $\vec{B}$ with the rotating axis aligning with the longitudinal direction of the tube was applied to induce the helical surface crawling locomotion. After the robot came into the slit, a 10-Hz counterclockwise rotating $\vec{B}$ was provided to generate the undulatory swimming to let the robot quickly go through the relatively straight gap. The robot’s swimming speed was observed to decrease in this shear-thinning fluid compared to a Newtonian fluid with a similar viscosity at low shear rates (fig. S14C). When the robot approached the outlet of the slit, we changed the rotating direction and frequency of $\vec{B}$ to clockwise and 1 Hz to achieve the undulatory crawling so that the robot could slowly poke the body out of the slit. After the robot fell into the tympanic cavity, a rotating $\vec{B}$ at the frequency of 10 Hz was lastly given to roll the robot away from the field of view.

## DISCUSSION

Here, the locomotion of the soft sheet-shaped millirobot in fluid-filled confined spaces is studied in detail in compared to our previous work realizing locomotion in dry confined regions (*18*). The constrained-space locomotion modes of the proposed sheet-shaped soft millirobot are dependent on the boundary geometry, the frictional property of the boundary surface, and the property of the fluid filling the space. Although the robot design is fixed, changing the magnetic field control inputs can enable different locomotion modes that are suitable for different fluid-filled constrained environments using a single soft-robot design. When facing a small gap that is highly curved, the undulatory crawling can achieve high maneuverability. When the small gap is filled with a viscous fluid, and its surface is smooth, the undulatory swimming is favorable to achieve effective locomotion. When the cross section of the working space is approximately a circle and has fluid flow inside, the helical surface crawling, which enables the robot to withstand the flow even if the external magnetic field is turned off, should be used. When moving in a big gap, the rolling motion or any other locomotion modes designed for open spaces can be adopted (*18*, *41*, *42*). Although the experiments conducted in this study are in 2D (two-dimensional), it is possible to steer the robot in 3D confined spaces by generating rotating magnetic fields in arbitrary planes in 3D space (*43*, *44*). The locomotion strategies proposed here could also be applied to sheet-shaped soft robots made of other smart materials (*45*–*47*) as long as they can produce the same deformations at the same boundary and fluid conditions.

To provide a guide to the design of the robot and the corresponding control signals, we summarize the relation between different quantifiable parameters and the locomotion speed in figs. S5 and S11, where the best quantifiable parameter combinations achieving the highest moving speed can be pinpointed. The strengths and weaknesses of each performance enhancement strategy can also be found in Table 1. Before exploiting these performance enhancement strategies, the strengths and weaknesses of each strategy must be weighed. For undulatory locomotion, enhancing the swimming speed by creating multiwavelength sinusoidal magnetization profiles would increase the robot size. Enhancing the crawling speed by adding spinules at both ends does not function well on hard smooth surfaces. Achieving better maneuverability by shortening the robot wavelength may result in a small length-to-thickness ratio (fig. S1) and cause a small deformation. For helical surface crawling, increasing the crawling speed and the stopping and drifting flow rates by creating grooves on the robot surface can increase the strength of the required magnetic field. Enhancing the capability to withstand the fluid flows by creating multiwavelength sinusoidal magnetization profiles would increase the robot size and make it hard to be twisted into the helical shape. Apart from the factors discussed above, the stiffness of the material used to fabricate the robot (table S2) also influences its locomotion speed. The results from both the experiments and simulations revealed that increasing the material stiffness will downgrade the performance of undulatory crawling, undulatory swimming, and helical surface crawling (fig. S15).

**Table 1:** Strengths and weaknesses of the soft robot performance and maneuverability improvement strategies.

***Locomotion mode***	***Performance enhancement strategy***	***Strength***	***Weakness***
**Body undulation**	Tuning δ	Higher crawling and swimming performance	Require prior knowledge about the size of the working environment
	Integrating spinules to both ends	Higher undulatory crawling performance	Not functioning on hard smooth surfaces
	Creating multiwavelength sinusoidal magnetization profile	Higher swimming performance	Larger robot size
	Shortening wavelength	Higher maneuverability	Too small deformation with too short wavelength
**Helical surface crawling**	Tuning γ	Increasing crawling speed; withstanding higher flow rate	Requiring prior knowledge about the size of the working environment
	Creating grooves	Increasing crawling speed; withstanding higher flow rates	Requiring larger magnetic torque
	Creating multiwavelength sinusoidal magnetization profile	Withstanding higher flow rates	Larger robot size; harder to twist into the helical shape

Although the soft-bodied locomotion makes the millirobot promising to navigate through tubular or slit-like lumen structures with cross-sectional dimensions at the millimeter scale inside the human body, such as the brain ventricles (*48*), cerebral aqueduct (*49*), urinary tract (*50*), bile ducts (*51*), Eustachian (*52*) and Fallopian tubes (*53*), and vascular systems (*35*), the influence of the in vivo environments on the robot locomotion performance must be carefully investigated. For example, the frictional properties of human tissue are different from the materials used to build the test environments in this paper (*54*), which may change the robot locomotion performance. In addition, many body fluids, such as mucus and blood, are non-Newtonian (*55*, *56*). Although the sheet-shaped robot has been demonstrated to be able to perform all four locomotion modes in a shear-thinning fluid (Fig. 7), how the shear-thinning property and the fluid viscoelasticity could influence the robot locomotion performance requires further study. The non-Newtonian behavior of the fluid can change the fluid force (*57*, *58*) acting on the soft body, affecting the robot performance by influencing the deformation amplitude and the propulsion produced. Moreover, the robot locomotion in this paper is designed in a forward design methodology, i.e., the robot design and the associated control signals were first generated by human knowledge and intuition and then verified in the experiments or simulations. An inverse design methodology can also be developed if the robot design and the associated control signals would automatically be generated by feeding the measured properties of the working environment, especially the boundary conditions and the fluid properties, to a dynamic locomotion model-based or a data-driven learning-based design algorithm. Such an automated design approach would enable the optimal exploitation of the adaptive multimodal locomotion modes for future biomedical applications.

## MATERIALS AND METHODS

### Soft millirobot fabrication

NdFeB magnetic microparticles (MQP-15-7, Magnequench; average diameter, 5 μm) were mixed with the uncured polymer (Ecoflex 00-10, Smooth-On Inc.) in a mass ratio of 1:1. The sample was degassed for 3 min and poured onto a preprepared flat acrylic substrate. A thin film of the polymer mixture was formed by running a razor blade across the surface to remove any excess mixture. The thickness of the film was controlled by the number of layers of the Scotch tape placed along the sides of the substrate. The polymer mixture was then cured at 65°C for 1 hour on a hot plate. The fabrication of the grooved robots was identical but involved the additional step of printing grooved features on the substrate before Scotch tape was applied to control the thickness of the film (Fig. 6C). To print the grooved features, the flat acrylic substrate was replaced by the silicon wafer with grooved features fabricated using two-photon polymerization (Photonic Professional GT, Nanoscribe GmbH) with the IP-S resin (Nanoscribe GmbH).

After the sample was cured, the thin film was cut in a LPKF ProtoLaser U3 laser cutter to the desired dimensions. The robots were then peeled off from the substrate with tweezers and wrapped around a suitably sized nonmagnetic rod depending on the length of the robot. Water-soluble glue was applied during this process to ensure that the robots formed a closed loop around the rod. The robots were then placed in a vibrating sample magnetometer and placed such that the openings where the robots were glued together were 45° to the direction of the magnetic field produced. This step introduced a phase shift to the sinusoidal magnetization profile when the robots were magnetized with a 1.8-T homogeneous magnetic field. The field was applied for 5 s. The robots were then detached from the rods and rinsed with deionized water (DI) until no more glue was observed on the surface.

For robots with multiwavelength sinusoidal magnetization profiles and robots with spinules, postfabrication assembly was required (fig. S6, A and B). Briefly, this involved applying uncured Ecoflex 00-10 as glue to join the structures together. In the former, the robot was joined with another robot of the same dimensions, while in the latter, the robot was glued to spinules. The concatenated structures were then left on a hot plate at 60°C for at least 3 min to allow the applied Ecoflex 00-10 to cure. The spinules were 3D-printed using two-photon polymerization with the IP-S resin.

### Friction measurements

Friction measurements were performed on a custom-made setup with a 25-g load cell (GSO-25, Transducer Techniques). Movement in the *x* direction was controlled by a piezo stage (LPS-65 200, Physik Instrumente GmbH & Co. KG). A customized LabVIEW code enabled control over the velocity and displacement in the *x* and *z* directions (fig. S13A). The sample had a test area of 4 mm by 1 mm and was visually checked to be parallel to the test surface before the measurements were taken. This sample was prepared as per the method stated above but was not magnetized. A preload of 8.88 mN was applied, and fluid was poured in before the *x* axis was translated at a rate of 0.01 mm/s. Before the voltage signals were transferred to the data acquisition board (PCIe-6259, National Instruments), the raw signals were first filtered through a signal conditioner (BNC-2110, National Instruments). The test substrates used for the measurements were 1:10 SYLGARD 184 silicone (polydimethylsiloxane, Dow Corning) and an stereolithography (SLA)–printed resin (BV-007A, MiiCraft). The used fluid was glycerol (VWR Chemicals).

### Preparation of the Newtonian and shear-thinning fluids

Glycerol (VWR Chemicals) and hyaluronic acid solutions (6 mg/ml) were used as the Newtonian fluid and shear-thinning fluid, respectively. The Newtonian fluid was prepared by mixing the glycerol and water with volume ratios of 1:1, 20:1, and 40:1 to produce fluid viscosities of 6, 343, and 720 cSt, respectively. The shear-thinning fluid was prepared by dissolving hyaluronic acid sodium salt (53747, Sigma-Aldrich) in phosphate-buffered saline (Gibco, Life Technologies). The solution was then stirred for 48 hours under room temperature to ensure that the salt had fully dissolved. The rheological properties of the fluids tested in this paper were measured by a TA Instruments Discovery HR-2 rheometer with a 20-mm parallel plate and a gap distance of 150 μm. All measurements were carried out at room temperature (20°C). At each shear rate, the fluid was given 5 s to stabilize before five readings were taken in a minute. The fluid used in each experiment can be found in table S1.

### Numerical simulations

To model the robot locomotion and fluid flow created by the robot, we used a computational fluid dynamics (CFD) model (*59*, *60*), which could accurately describe the robot motion and the deformation-induced fluid flow at low *Re*. In the CFD model, the midplane of the robot was meshed by shell elements. The membrane and the bending stiffness were considered by using constant strain triangles with drilling degrees of freedom (*61*) and triangular Kirchhoff elements (*62*), respectively. The large and geometrically nonlinear deformation of the soft robot was modeled by adopting an updated Lagrangian approach. To compute the fluid flow generated by the soft robot motion, the boundary element method was used. The Stokes equation was used to model the fluid flow, the solution of which can be written by Green’s functions in free space. The drag forces on the robot surface were treated as a distribution of point forces. The velocity ***u*** at a point in the fluid regime with position ***r*** due to a point force exerted by the node on the robot body at position ***r***′ could be obtained as

$$u=Gf\;\text{and}\;G_{\mathrm{ij}}=\frac{1}{8\pi \mu }\{\frac{\delta_{\mathrm{ij}}}{R}+\frac{R_{i}R_{j}}{R^{3}}\}\;(i=1,2,3)$$ (1)

with ***R*** = *r* − ***r***′, *R* =∣***R***∣, δ*_ij_* is the Kronecker delta, and ***G*** is the Green’s function for a point force ***f***(***r***′) acting in a fluid. These point forces (***f*** = ***T****d*S, where ***T*** is the robot surface traction to the fluid) were assumed to be distributed over the surface of the robot and assumed to vary linearly over each triangular surface element, resulting in the fluid velocity

$$u=\sum_{\text{nelm}}\int_{S}\text{GT}\mathrm{dS}=\sum_{\text{nelm}}\int_{S}\text{GN}\mathrm{dS}t$$ (2)

where *nelm* is the number of surface elements and the tractions were linearly interpolated using ***T*** = ***Nt*** with ***t*** being the tractions at the nodes. Equation 2 holds at every point in the fluid, including the nodes on the robot surface ***u****^r^*. By assembling these equations in a matrix ***G****^r^*, i.e., ***u****^r^*= ***G****^r^****t***, we could get the traction exerted by the robot on the fluid by inverting this relation ***t*** = (***G****^r^*)^−1^***u***^r^. Then, the fluid velocity induced by the robot in the fluid domain could be obtained by substituting the tractions ***t*** into Eq. 2.

For undulatory locomotion, the virtual robot was immersed in the middle area of the fluid domain, which was represented by a rectangular channel (fig. S3A). The no-slip boundary condition was implemented to all channel walls. To model the contact between the robot and the wall, we used the constraint normal force and friction, which included static friction and sliding friction. The normalized jet flow along the longitudinal direction of the channel, $u_{x}^{n}(t)$, was calculated within the chosen volume (the red shaded area marked in fig. S3A) at a particular time instant *t* to quantify the robot’s ability to transport the fluid. It is defined as

$$u_{x}^{n}(t)=\frac{u_{x}(t)}{{\text{max}}_{0\leq t\leq T}(|u_{x}(t)|)}$$ (3)

with

$$u_{x}(t)=\frac{1}{z_{\text{max}}-z_{\text{min}}}\int_{z_{\text{min}}}^{z_{\text{max}}}[\frac{1}{4\varepsilon \delta }\int_{x_{0}-\varepsilon }^{x_{0}+\varepsilon }\int_{y_{0}-\delta }^{y_{0}+\delta }u_{x}(x,y,z,t)d\mathrm{xdy}]dz$$ (4)

where *T* is the time period of one undulation cycle. *z*_max_ and *z*_min_ are the heights of the upper and the bottom channel walls, respectively. ε and δ are the thickness and width of the calculation volume, respectively, and *x*_0_ and *y*_0_ denote the position of the calculation volume, which are 5.5 and 0 mm, respectively.

For helical surface crawling, the robot was placed in a virtual tube (fig. S3B). The heterogeneous sliding friction for helical surface crawling was formulated as the sum of the longitudinal and transverse friction (fig. S7) as ***f***_sum_ = ***f***_∥_ + ***f***_⊥_, with ***f***_∥_ = μ_∥_***u***_∥_ and ***f***_⊥_ = μ_⊥_***u***_⊥_ (*63*). The transverse friction coefficient was chosen larger than the longitudinal one in the helical surface crawling mode, i.e., μ_⊥_ > μ_∥_.

### Force analysis of helical surface crawling

During helical surface crawling, the robot simultaneously conducts a translational locomotion along the tube and a rotational locomotion relative to the centerline of the tube. For ease of discussion, we assumed that the robot was always at the quasi-static state during locomotion. We projected the robot onto the inner wall of the tube and expanded the tube wall into a 2D plane. In this way, the rotational locomotion can be represented by the translational movement along the *y* direction, and the actuation force ***F***_mag_ provided by the magnetic field can be expressed as a constant vector in the *y* direction (fig. S10A). During the steady crawling state, the whole robot body that translates with a velocity ***u***_sum_. ***u***_sum_ can be further decomposed as ***u***_∥_ and ***u***_⊥_ that induce the friction forces ***f***_∥_ and ***f***_⊥_ along the longitudinal and transverse directions, respectively. The resultant friction ***f***_sum_ is the vector sum of ***f***_∥_ and ***f***_⊥_, and its components along the *x* and *y* directions, ***f****_x_* and ***f****_y_*, balance the drag force ***F***_drag_ and ***F***_mag_, respectively. ***F***_drag_ is the drag that prevents the forward crawling of the robot. Comparing to the case of twisting a screw, the difference here is the existence of the slip velocity along the transverse direction (***v***_⊥_). If the grooves are created along the longitudinal direction of the robot at this equilibrium state, then μ_⊥_ increases more than μ_∥_ (fig. S13B), i.e., the frictional anisotropy is enhanced. Assuming that γ is not changed by changing μ_⊥_ and μ_∥_, the pressure acting on the robot is unchanged. As a result, ∣***f***_⊥_∣ and ∣***f***_∥_∣ increases to ∣***f***′_⊥_∣ and ∣***f***′_∥_∣, respectively. This breaks the equilibrium state by accelerating the robot in the −*x* direction (∣***f****_x_*∣ increases to ∣***f***′*_x_*∣) in view of the pitch angle α that is always smaller than 90° at this helical shape (fig. S10B). However, because the increase of ∣***f***_∥_∣ and ∣***f***_⊥_∣ also increases ∣***f****_y_*∣, a larger ∣***F***_mag_∣ is required to actuate the robot.

## SUPPLEMENTARY MATERIALS

Supplementary material for this article is available at http://advances.sciencemag.org/cgi/content/full/7/27/eabh2022/DC1
